# Endoscopic Submucosal Dissection for Treatment of Patients Aged 75 Years and over with Esophageal Cancer

**DOI:** 10.5402/2012/671324

**Published:** 2012-06-17

**Authors:** Osamu Kikuchi, Hirokazu Mouri, Kazuhiro Matsueda, Hiroshi Yamamoto

**Affiliations:** Department of Gastroenterology, Kurashiki Central Hospital, 1-1-1 Miwa, Kurashiki 710-8602, Japan

## Abstract

*Background*. Although many reports concerning the use of endoscopic submucosal dissection (ESD) for esophageal cancer have been published, the feasibility of ESD in elderly patients has not been reported. Therefore, we evaluated the efficacy and safety of ESD for treating early esophageal cancer in elderly patients. *Methods*. A total of 62 cases (52 men, 10 women; mean age ± standard deviation, 66.5 ± 10.5 years) for which the first resection (first treatment) of esophageal cancer was performed by ESD were identified from 77 consecutive esophageal epithelial cancers in 67 patients treated at our institution from January 2005 to March 2011. Patient characteristics, clinical findings, and outcomes were retrospectively assessed for patients separated into older (aged 75 years and older) and younger (aged under 75 years) groups. *Results*. No significant differences in specimen size, procedure time, median length of the hospital stay (8 versus 9 days; *P* = 0.252) or procedure-associated complications (8% versus 27%; *P* = 0.264) were observed between the older (*n* = 13) and younger (*n* = 49) groups. Lesions were completely resected in 12 patients and 44 patients, in the younger and older groups, respectively, and the curative resection rate was 77% and 59%, respectively. There were no deaths attributable to procedure-associated complications. *Conclusions*. ESD is an effective treatment for early esophageal cancer and is well tolerated by elderly patients.

## 1. Introduction

The Japanese population is rapidly aging, and approximately 30% of all esophageal cancer patients are 75 years of age or older [1]. Endoscopic mucosal resection (EMR) has been suggested to produce satisfactory outcomes [2, 3] and is widely performed for early cancers of the esophagus. However, it is difficult to perform en bloc resection using EMR when dealing with lesions over 20 mm in size. Piecemeal resection of lesions can interfere with precise histological examination, in particular, making identification of residual lesions and local recurrence difficult. In order to address this issue, endoscopic submucosal dissection (ESD) has been recently developed for use in early gastric cancer [4–7] and has also become an effective treatment for early esophageal cancer [8, 9]. However, because the wall of the esophagus is thinner and the lumen more narrow compared with the stomach, this procedure is thought to be associated with technical difficulties and, as a consequence, a higher incidence of complications than conventional EMR.

Elderly patients with esophageal cancer often have more comorbidities and lower performance status than do nonelderly patients. Therefore, treatments for these patients must be considered in this context. Although many reports concerning the use of ESD for esophageal cancer have been published, the feasibility of ESD in elderly patients with esophageal cancer has not been reported.

We retrospectively reviewed our ESD cases to evaluate the efficacy and safety of ESD in patients aged 75 years or older with early esophageal cancer.

## 2. Methods

### 2.1. Subjects

From January 2005 to March 2011, we identified a total of 77 consecutive esophageal epithelial cancers in 67 patients treated with ESD from a database of endoscopic resection. The database included data on demographics, endoscopic diagnosis, biopsy results before treatment, and pathological treatment results. We retrospectively assessed 62 procedures in 62 patients as first treatment for esophageal cancer (52 men, 10 women; mean age ± standard deviation (SD) 66.5 ± 10.5 years). We excluded 15 procedures because patients had been previously treated for esophageal cancer with ESD or other treatment.

The patients were divided into 2 groups: the older group, containing all patients 75 years and older, and the younger group, consisting of all patients under 75 years. We reviewed medical records including physical examination, standard laboratory tests, chest radiograph, upper gastrointestinal (GI) endoscopy, and computed tomography (CT) of the neck, chest, and abdomen to collect demographic data, and data on complications and length of hospital stay after ESD. All patients provided written informed consent for the ESD procedure. The protocol of this study was approved by the institutional review board of Kurashiki Central Hospital.

### 2.2. ESD Procedure

ESD was indicated if the esophageal tumor had a superficial morphology and was clinically assessed as noninvasive carcinoma or invasive carcinoma confined to the mucosa or upper third of the submucosa based on endoscopic findings. Antiplatelet agents were usually stopped for 3 to 7 days before treatment. Patients were sedated by intravenous administration of pethidine and diazepam. ESD was performed using a conventional single-channel endoscope (GIF-H260Z, −Q260J, or −Q260; Olympus, Tokyo, Japan) or a 2-channel endoscope (GIF-2TQ260M, Olympus). Prior to resection, lesions were stained with iodine to identify the margin. Several spots were marked 3–5 mm outside the margin of each lesion by using argon plasma coagulation or Flush knife (DK2618JB or DK2618JN, FTS, Tokyo, Japan). After injection of 1% hyaluronic acid (Suvenyl; Chugai Pharmaceutical Co., Tokyo, Japan) or 0.4% hyaluronic acid (MucoUp; Seikagaku Kogyo Co., Tokyo, Japan) mixed with 10% glycerin plus 5% fructose and 0.9% saline preparation (Glyceol; Chugai Pharmaceutical Co.) into the submucosa, we performed mucosal incision and submucosal dissection. A small amount of indigo carmine dye and epinephrine was also mixed with this solution to allow the injected area to be distinguished from the noninjected area. The ESD procedure was mainly performed with Flex knife (KD-630L, Olympus; 2006 and before) and Flush knife (2007 and after), and we also used Mucosectome (DP2518, Pentax, Tokyo, Japan) as necessary. Injection of hyaluronic acid solution was repeated as needed, and further resection was carried out to ensure total removal of the lesion. To control bleeding, hemostatic forceps (SDB2422, Pentax, or Coagrasper: FD-410LR, Olympus) were used as necessary. We used metallic clips (EZ clip: HX-610-090L and HX-610-135; Olympus) to close exposures of the muscular layer larger than approximately 3 mm and, in 1 case, a Mallory-Weiss tear. Carbon dioxide insufflation was used for ESD procedures after October 2008. Cardiorespiratory function was continually monitored throughout the procedure.

Endoscopic reexamination was performed 5–7 days after treatment to rule out complications such as bleeding and perforation. Chest radiograph was performed immediately after ESD or the next day to evaluate if perforation or mediastinal/subcutaneous emphysema had occurred. All ESDs were performed or supervised by endoscopists board certified by the Japanese Gastroenterological Endoscopy Society.

Intraoperative hemorrhage was defined as bleeding that was not controlled promptly with electrocautery knives or hemostatic forceps. Postoperative hemorrhage was defined as the occurrence of hematemesis or tarry stool with endoscopic confirmation of exposed vessels or bleeding.

### 2.3. Evaluations

Histological type, size, depth of invasion (m1, m2, m3, sm1, sm2), lateral and vertical margins, and lymphatic-vascular invasion were evaluated according to the Japanese Classification of Esophageal Carcinoma [10]. The approximate area of the specimen was calculated (area = *π* × length/2 × width/2). Definitions used for the evaluation of therapeutic outcomes were as follows: complete resection was defined as en bloc resection with tumor-free margins; curative resection was defined as complete resection with depth of m1 or m2 (confined to lamina propria mucosae) and without lymphatic or venous invasion. If the treatment was not curative, we recommended additional treatment such as esophagectomy or chemoradiotherapy.

### 2.4. Statistical Methods

All data were analyzed by using IBM SPSS statistics, version 19 (SPSS Inc., Chicago, IL, USA). Statistical analyses were performed using Fisher's exact test and the Mann-Whitney *U*-test. A two-tailed *P* value of <0.05 was considered statistically significant.

## 3. Results

Thirteen of the 62 patients were ≥75 years of age (older group), and 49 were <75 years of age (younger group). Baseline characteristics of patients are shown in Table 1. The incidence of major comorbidities was significantly higher in the older group than in the younger group (77% (10/13) versus 31% (15/49), resp.; *P* = 0.003). The most frequent comorbidity was a history of pulmonary conditions (present in four and six patients in the older and younger groups, resp.). American Society of Anesthesiology (ASA) classification scores were significantly higher in the older group than in the younger group (*P* = 0.007).

Thirteen patients (100%) in the older group and 46 (94%) in the younger group had squamous cell carcinoma. The location of the tumor did not significantly differ between the two groups (*P* = 0.114) (Table 2).

There were no significant differences in specimen size, procedure time, or proportion of carbon dioxide insufflation between the two groups. With regard to pathological evaluation after ESD, 11 patients were m1-m2, 2 were m3-sm1, and 0 were sm2 in the older group, and 33 were m1-m2, 12 m3-sm1, and 4 sm2 in the younger group. The complete resection rates were 92% (12/13) and 90% (44/49) in the older and younger groups, respectively. The curative resection rates were 77% (10/13) and 59% (29/49) in the older and younger groups, respectively (Table 3). Furthermore, there were no significant differences in the complete and curative resection rates between the two groups (*P* = 1.000, 0.338, resp.)

The ESD-associated complication rates did not significantly differ between the older and younger groups (8% (1/13) versus 27% (13/49); *P* = 0.264). The most frequent complication was mediastinal emphysema without obvious perforation (0 and 8 in the older and younger groups, resp.). We observed two cases of bleeding (one Mallory-Weiss tear during ESD, one hemorrhagic esophageal ulcer 7 days after ESD). Conservative therapy resolved all complications. The median length of the hospital stay after ESD did not differ significantly between the older and younger groups (8 days versus 9 days; *P* = 0.252) (Table 4). No deaths were attributable to ESD-associated complications.

## 4. Discussion

Our study examined the efficacy and safety of ESD for esophageal cancer in elderly patients. The need for treatment of esophageal cancer among elderly patients is increasing. Most elderly patients have one or more physical or mental comorbidities that may affect treatment strategies. In this study, 77% of elderly patients ≥75 years old had some comorbidities, and elderly patients had significantly higher ASA classification scores than younger patients. However, elderly patients should still receive curative treatment to prevent disease progression as long as comorbidities can be adequately managed.

EMR is established as a curative and safe treatment for early esophageal cancer, and good treatment results have been reported using this technique. However, en bloc resection of lesions >2 cm in diameter using EMR is difficult and may also create difficulties in histological evaluation of resected tissue.

ESD is now widely performed for esophageal tumors with high en bloc resection rates [8, 9]. In this study, we observed a high rate of endoscopic en bloc resection both in the older group (92%) and the younger group (90%), which is approximately equivalent to that stated in previous reports [8, 9]. Thus, ESD is effective treatment for early esophageal cancer not only in younger patients but also in elderly patients.

The complication rate of ESD is thought to be higher than that of EMR because of technical issues and relatively long procedure times, which are potential concerns in elderly patients who often have comorbidities such as ischemic heart disease, stroke, pulmonary emphysema, and diabetes mellitus. In our study, there were 8 cases (13%) of mediastinal/subcutaneous emphysema. This relatively higher proportion compared with previous reports [11, 12] was thought to be mainly due to the manner and timing of diagnosis. As mentioned above, we performed chest radiograph immediately following ESD in some cases. Tamiya et al. observed a 31% rate of mediastinal emphysema as detected by chest CT immediately following ESD compared with only 1.7% by chest radiograph [13]. Maeda et al. observed a 63% rate of mediastinal emphysema diagnosed by multidetector CT immediately following ESD compared with only 6.6% with chest radiograph performed on the day following ESD [14]. In these studies, mediastinal emphysema cases detected only by CT were not deemed to require clinical intervention except for antibiotics. In fact, we treated all 8 cases conservatively and all recovered. Another possible reason was the comparatively lower proportion of carbon dioxide insufflation in the younger group, although this difference was not statistically significant due to the small patient population. The other complications, including pneumothorax, mediastinitis, bleeding, and strictures, were also managed successfully with conservative or endoscopic treatment without any sequelae.

Hospital stay following ESD was similar in both groups. Consequently, we believe ESD can be used in elderly patients with equivalent cost.

ESD may have advantages not only for en bloc resection but also for precise pathological diagnosis. There were 2 cases in elderly patients found to be m3-sm1 based on ESD. In the Japanese guidelines, ESD is definitely indicated in m1-m2 and relatively indicated in m3-sm1, the latter because there is possibility of lymph node metastasis if the invasion depth is more than m3 [15]. However, it has been reported that evaluating the invasive depth of superficial esophageal cancer on the basis of endoscopic findings before resection was accurate in only approximately 80% of cases [16]. Endoscopic ultrasound has limitations [17, 18]. Thus, we believe that ESD should be performed when the tumor is thought to be m1-sm1 based on endoscopic evaluation, and additional treatment should be considered when the resection is noncurative according to pathological evaluation of tumor depth following ESD. Using this strategy, we may avoid more invasive, unnecessary treatment such as surgery or radiation therapy, at least in some cases.

Our study has limitations. These include retrospective design, lack of long-term observation, and the small numbers of patients with each complication.

In conclusion, we found that en bloc resection rate was similar and the prevalence of ESD-associated complications was not significantly increased among elderly patients. Thus, ESD appears to be effective against early esophageal cancer and is well tolerated by elderly patients. 

## Figures and Tables

**Table 1:** Demographics and baseline characteristics.

	≥75 years (*n* = 13)	<75 years (*n* = 49)	*P* value
Gender (M/F)			n.s.
Female	2	8	
Male	11	41	
Age (years : median, range)	79 (76–87)	65 (42–74)	<0.001
Any comorbidities	10 (77%)	15 (31%)	0.004
Cardiovascular disease	2 (15%)	6 (12%)	
Pulmonary disease	4 (31%)	6 (12%)	
Diabetes mellitus	2 (15%)	4 (8%)	
Liver cirrhosis	0 (0%)	1 (2%)	
Renal insufficiency	0 (0%)	0 (0%)	
Cerebrovascular disease	1 (8%)	1 (2%)	
Other	1 (8%)^∗^	0 (0%)	
ASA classification			0.019
1	3	32	
2	7	13	
3	3	4	
4	0	0	

^∗^One case of idiopathic thrombocytopenic purpura.

n.s.: not significant.

ASA: American Society of Anesthesiology.

**Table 2:** Histological type and tumor location.

	≥75 years (*n* = 13)	<75 years (*n* = 49)	*P* value
Histological type			n.s.
Squamous cell carcinoma	13 (100%)	46 (94%)	
Adenocarcinoma	0 (0%)	3 (6%)	
Tumor location			n.s.
Cervical	0 (0%)	0 (0%)	
Upper thoracic	3 (23%)	5 (10%)	
Mid-thoracic	9 (69%)	33 (67%)	
Lower thoracic	1 (8%)	8 (16%)	
Esophagogastric junction	0 (0%)	3 (6%)	

n.s.: not significant.

**Table 3:** Treatment results.

	≥75 years (*n* = 13)	<75 years (*n* = 49)	*P *value
Area of specimen (cm^2^ : median, range)	6.9 (3.9–8.0)	6.6 (1.3–23.8)	n.s.
Procedure time (min : median, range)	107 (54–187)	127 (42–485)	n.s.
CO_2_ insufflation used	10 (77%)	24 (49%)	n.s.
Depth of tumor invasion			n.s.
m1-m2	11	33	
m3-sm1	2	12	
sm2	0	4	
Horizontal margin			n.s.
pHM0	12	45	
pHMXor1	1	4	
Vertical margin			n.s.
pVM0	13	49	
pVMXor1	0	0	
Complete resection	12 (92%)	44 (90%)	n.s.
Curative resection	10 (77%)	29 (59%)	n.s.

m1: intraepithelial carcinoma; m2: microinvasive carcinoma (invasion through the basement membrane); m3: intramucosal carcinoma (invasion to the muscularis mucosae); sm1: superficial invasion in the submucosa (extended up to 200 mm below the lower border of the lamina muscularis mucosae); sm2: deep invasion in the submucosa (deeper than 200 mm below the lower border of the lamina muscularis).

complete resection: en bloc resection with tumor-free margins; curative resection: complete resection with depth of m1 or m2 and without lymphatic or venous invasion.

n.s.=not significant.

**Table 4:** Complications and hospital stay.

	≥75 years (*n* = 13)	<75 years (*n* = 49)	*P *value
Any complication	1 (8%)	13 (27%)	n.s.
Mediastinal/subcutaneous emphysema	0 (0%)	8 (16%)	
Pneumothorax	0 (0%)	1 (2%)	
Mediastinitis	1 (8%)	2 (4%)	
Major perforation	0 (0%)	0 (0%)	
Bleeding	0 (0%)	2 (4%)	
Stricture	0 (0%)	2 (4%)	
Hospital stay after ESD (days : median, range)	8 (6–14)	9 (6–16)	n.s.

n.s.=not significant.
